# Gamma In Addition to Neutron Tomography (GIANT) at the NECTAR instrument

**DOI:** 10.1038/s41598-023-47237-y

**Published:** 2023-11-17

**Authors:** Richi Kumar, Lucas Sommer, Anton S. Tremsin, Adrian S. Losko

**Affiliations:** 1 German Engineering Materials Science Centre (GEMS) at Heinz Maier-Leibnitz Zentrum (MLZ), Helmholtz-Zentrum Hereon, Lichtenbergstr.1, 85748 Garching, Germany; 2 Heinz Maier-Leibnitz Zentrum (MLZ), Technical University of Munich, Lichtenbergstr. 1, 85748 Garching, Germany; 3 Space Sciences Laboratory, University of California, Berkeley, CA 94720 USA

**Keywords:** Techniques and instrumentation, Imaging techniques, Techniques and instrumentation, Imaging techniques

## Abstract

The NECTAR instrument provides access to thermal and fast neutrons which are suitable for non-destructive inspection of large and dense objects. Scintillators are used in combination with a camera system for radiography and tomography. Gamma-rays are produced as inevitable by-products of the neutron production. Furthermore, these gamma-rays are highly directional due to their constraint to the same beam-line geometry and come with similar divergence as the neutrons. We demonstrate how these gamma-rays, previously treated as beam contamination can be used as a complementary probe. While difficult to shield, it is possible to utilize them by using gamma sensitive scintillator screens in place of the neutron sensitive scintillators, viewed by the same camera based detector system. The combination of multiple probes often provides complementary information that can result in a better contrast or insight into the sample composition, for a broader range of materials and applications. Hence dual-mode imaging, combining thermal/cold neutrons with X-ray imaging has been developed at many neutron facilities. With X-rays limited in penetration of dense materials to millimeters only, we present a multimodal imaging technique that is capable of penetrating cm-sized objects using thermal to fast neutrons with the addition of gamma-rays by changing the combination of scintillator and beam filter used at the NECTAR instrument.

## Introduction

Radiography and tomography using X-rays, gamma-rays and neutrons have found application in wide-ranging scientific fields^1–5^. The unique ability of these particles/photons to penetrate through objects and enable non-destructive imaging has made them indispensable characterization tools. Among these, neutrons and photons are considered complementary probes due to the fundamental difference in the nature of their interactions with matter^6,7^.

Upon penetration, photons interact mainly by photoelectric absorption, Compton scattering or pair production depending on their energy and the atomic number of the material^8,9^. In contrast, neutrons interact with the nucleus, providing vastly different contrast mechanisms^10^. The interaction cross sections depend heavily on the energy of incoming neutrons and are not only element but also isotope-specific. Neutrons are loosely categorized based on their energy $E_{n}$ as cold with $0.005\;{\text{ meV}}\; \le E_{n} \; < \;0.010 \;{\text{eV}}$, thermal with $E_{n} \cong 0.025 \;{\text{eV}}$, epithermal with $\sim 1 eV < E_{n} \le 100\;{\text{ keV}}$, and fast with $E_{n} > 1\; {\text{MeV}}$^11^. In general, it can be noted that thermal neutrons have a shorter attenuation length as compared to fast neutrons, owing to the higher kinetic energy of fast neutron.

Neutrons provide good contrast to several light elements—specifically for hydrogen and lithium—enabling many key characterizations that are not possible with photons. For example, neutron imaging is often used in in situ and in operando studies of fuel cells and batteries^12,13^. In case of bulky objects in situ fast neutron radiography provided unique insights into the distribution of hydrogen in scaled-up metal hydride hydrogen storage system^14^. Here, conventional X-ray imaging would fail to provide sufficient contrast for hydrogen and sufficient transmission through heavy metals.

Because of the vastly different contrast mechanism of photons and neutrons, it is crucial to choose the optimal probe for an investigation. There are many examples that would strongly benefit from a combination of both techniques. For example, if water intake in a concrete block is to be studied with respect to its pore size, neutron imaging enables imaging of the water due to the high neutron cross section of hydrogen, while the pores can be imaged using X-rays, which is well suited for high resolution imaging of the pores or defects^15^.

A combination of cold and thermal neutron imaging with X-ray imaging has been established at different large-scale neutron facilities^6,7,16,17^. As such, the case for multimodal imaging is well established and having the capability to characterize samples using different probes indisputably enhances investigation capabilities. The combination of cold neutrons and X-rays has limitations in sample size (< 1 cm), hence a combination of thermal and fast neutron imaging along with gamma-ray imaging is needed for investigation of larger samples (> 1 cm) and in the presence of large sample environments such as autoclaves and furnaces.

At NECTAR, two neutron spectra dominated by either fast or mixed: thermal plus fast neutron energies can be used for imaging. In both cases, gamma-rays are present in the radiation field. All three components of the beam can be utilized to perform multimodal imaging with very little change to the existing setup. This has been implemented at the NECTAR instrument as Gamma in Addition to Neutron Tomography (GIANT). In the following sections, the details about the instrument setup needed to realize such imaging capabilities and first proof of concept measurements are provided.

## Experimental setup at NECTAR

The NECTAR instrument is a unique large scale instrument which was designed primarily for fast neutron imaging^18–20^. It is located at the SR10 beam port of the research reactor FRM II^19^. A set of movable enriched ^235^U converter plates at the tip of the beam port can be used to convert the moderated thermal neutrons from the reactor pool into fission neutrons. Gamma-rays are also generated as a by-product of the production of fission neutrons^21^. The incoming fission beam at NECTAR constitutes a mixed spectrum of neutrons and gamma-rays from fission reactions, accompanied by thermal and epithermal neutrons.

**Figure 1:**
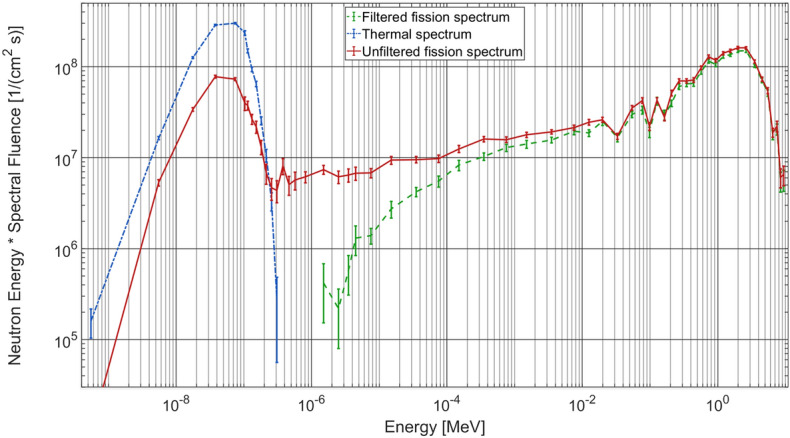
MCNP calculated energy distribution of the neutron flux at NECTAR, for thermal spectrum (without converter plate, no filter) in blue, unfiltered fission spectrum (with converter plate, no filter) in red and filtered fission spectrum (with converter plate and Cd-B filter) in green.

Three neutron spectra at NECTAR calculated using the Monte Carlo code MCNP 6.2 at a distance of 528 cm away from the converter plates are shown in Fig. 1 (see section “MCNP calculations” for details). The thermal spectrum without the enriched ^235^U converter plates is shown in blue. The unfiltered fission spectrum with the converter plate is given in red. For fast neutron imaging, a Cd-B filter (2 mm Cadmium and 10 mm Borated rubber) is used along with the converter plate to absorb the remaining thermal neutrons, resulting spectrum is shown in green.

The average prompt gamma-ray energy generated in fission reactions in the converter plates is ~ 1 MeV^22^. Additional interactions of neutrons with materials in the beam result in secondary gamma-rays contributing to the gamma spectrum at NECTAR. As a rough estimate, the total gamma flux at NECTAR is estimated to be equal to that of the fast neutron flux. To reduce gamma-ray contributions in neutron imaging, scintillators that are non-sensitive to gamma-rays, like those made of Boron or Lithium^23^, are typically utilized.

The detection of particles with scintillators is a two-step process, where particles are first converted to visible light using a scintillating material. Consequently, a camera is used to detect the visible light emitted by the scintillator. Since fast neutrons generally provide low attenuation for most materials, including those of the detector itself, detection efficiencies for fast neutron scintillators are low (<< 10%). To increase the detection efficiency, composite scintillators consisting of hydrogen-rich polypropylene (PP) and ZnS are often the scintillators of choice at the NECTAR instrument^23^.

### GIANT at NECTAR

As seen in the previous section, different neutron spectra accompanied with gamma-rays are available at NECTAR (Fig. 1). For typical fast neutron imaging experiments, anything apart from fast neutrons is considered undesirable background. However, it is possible to use these erstwhile “backgrounds” to obtain additional contrast. Here, we demonstrate how these generally unwanted radiation of the beam can be used for multimodal imaging at the instrument.

**Figure 2:**
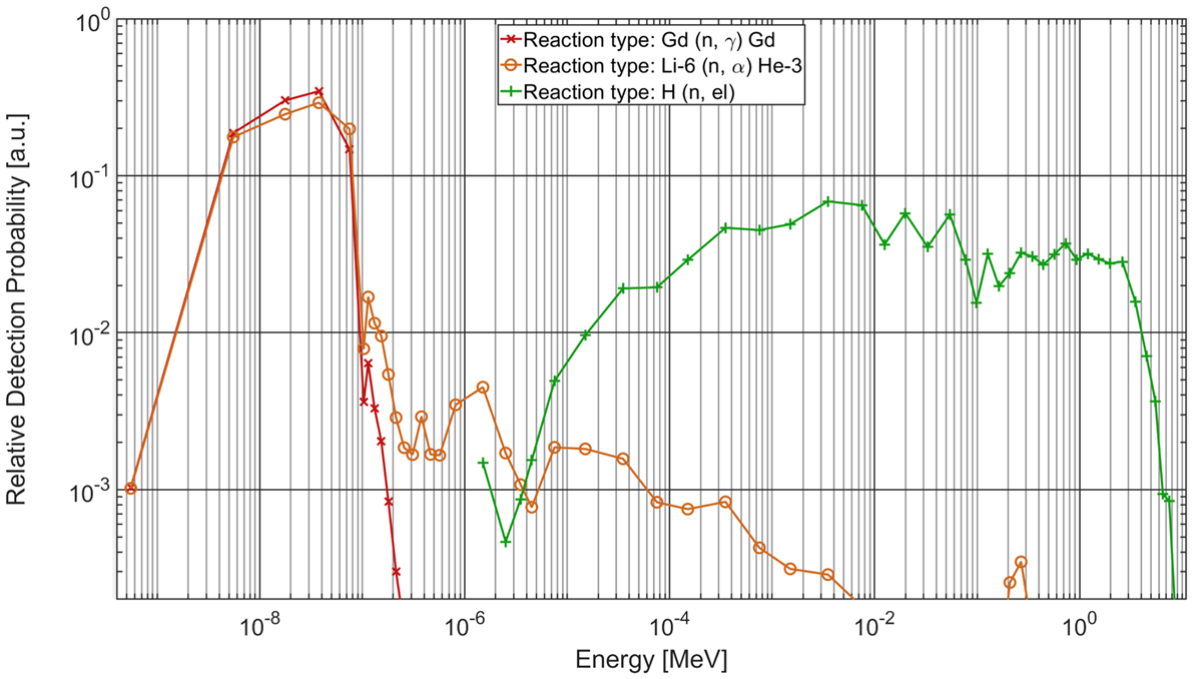
The relative detection probability of the neutron spectrum at NECTAR for different scintillation materials.

GIANT multimodal imaging involves imaging using a single setup allowing remote changing of the combination of scintillators and filters in the beam path. These combinations of scintillators and filters corresponding to the different imaging modalities are addressed as three Configurations in the manuscript. As such, fast neutron imaging is performed by utilizing a PP/ZnS:Ag scintillator with Cd-B filter using the typical detection setup as described in the previous section (Configuration #1). Figure 2 shows the relative detection probability over neutron energy of different scintillation materials for relevant neutron spectrum (details about the detection probability calculations are provided in section “Relative detection probability calculations”). The green plot in Fig. 2 shows that fast neutrons show a high detection probability for a hydrogenous PP/ZnS:Ag scintillator using the filtered fission spectrum (green plot in Fig. 1). It should be noted that during fast neutron imaging, a non-insignificant gamma-ray contribution is always present. Pb filters can be used to suppress these contributions, particularly lower energy gamma-rays, however the gamma-rays emitted from fission conversion, such as at NECTAR, typically have energies in MeV range and require several centimeters of Pb to be fully suppressed^23^. Therefore in the process of filtering the gamma-rays, the neutron flux would also be significantly reduced.

For thermal neutron imaging the same setup is used with a Gadox scintillator (Gadox is Gadolinium Oxysulfide doped with Terbium or Gd_2_O_2_S:Tb) but without Cd-B filter **(**Configuration #2). The detection probability for Gadox in combination with the unfiltered fission spectrum is indicated in red in Fig. 2. Due to the inherently low neutron cross section of Gd for fast neutrons, this scintillator primarily interacts with thermal neutrons present in the incoming beam. However, it is known that Gadox scintillators are sensitive to both thermal neutrons as well as gamma-rays. Hence, they enable imaging with a combination of thermal neutrons and gamma-rays. The orange plot in Fig. 2 shows similar interaction behavior for Li-6, albeit Li being a light element does not interact with gamma-rays significantly. Hence, another common scintillator, namely ZnS:^6^LiF, can be used instead of the Gadox scintillator if a higher suppression of gamma-ray detection for thermal neutron imaging is desired.

Further, using the Gadox scintillator with Cd-B filter enables suppression of thermal neutrons present in the direct beam, allowing for gamma-ray imaging **(**Configuration #3). It should be noted that while a Cd-B filter will primarily suppress thermal neutrons, it would inevitably also reduce gamma-ray flux. However, this would be insignificant for high energies gamma rays and hence can be ignored.

As a result, by interchanging the scintillator and filter configuration at NECTAR, multimodal imaging can be performed. The details are summarized in Table 1. Using this approach, both radiography and tomography are possible. Without the need to move the sample or the imaging camera, the resulting images for different modalities are automatically co-aligned. In the following section, a proof of concept of this technique is demonstrated.

**Table 1:** Configuration of filter, scintillators and corresponding NECTAR spectrum used for GIANT.

Configuration	Type of imaging	Filter	Scintillator	Corresponding NECTAR spectrum
#1	Fast neutron imaging	Cd-B	PP/ZnS:Ag	Filtered fission spectrum (Green spectrum in Fig. 1)
#2	Thermal neutron plus Gamma-ray imaging	–	Gadox	Unfiltered fission spectrum (Red spectrum in Fig. 1)
#3	Gamma-ray imaging	Cd-B	Gadox	Filtered fission spectrum (Green spectrum in Fig. 1)

## Qualitative assessment using different scintillators

### Step-wedges

**Figure 3:**
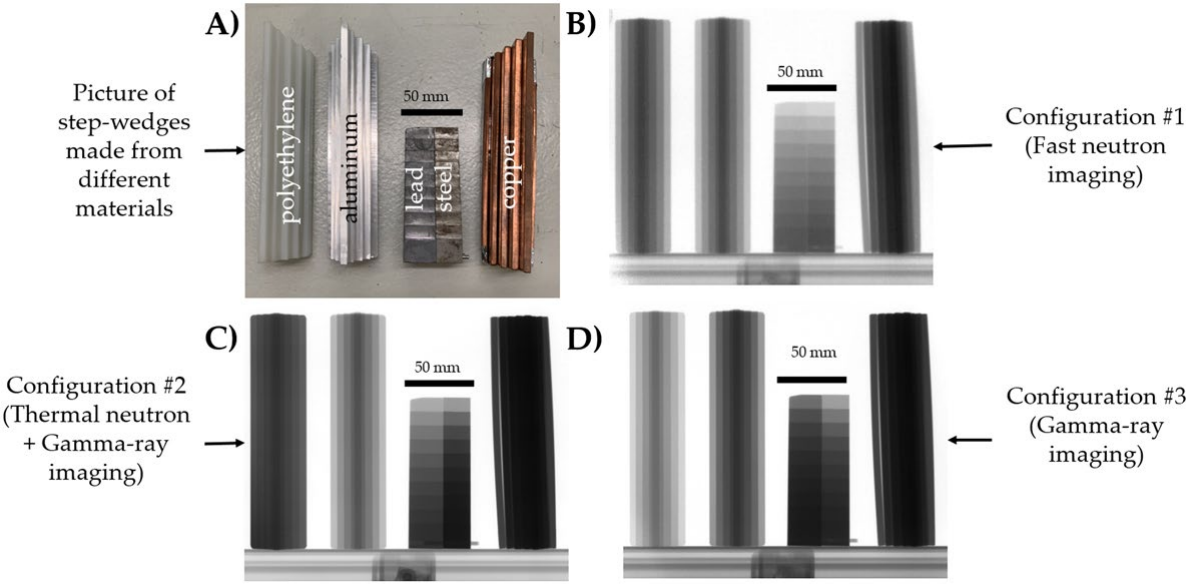
(**A**) Picture of step-wedges made of different materials that have been marked. Normalized radiograph of the step-wedges obtained using the different imaging modalities, (**B**) Configuration #1, (**C**) Configuration #2, (**D**) Configuration #3. Refer to Table 1 for details about the Configurations.

To demonstrate GIANT multimodal imaging, step wedges of different materials ranging from plastic (Polyethylene: PE) to light metal: Aluminum (Al) and heavier metals/alloy: Steel, Copper (Cu) and Lead (Pb), were imaged using the approach described in the previous section. Figure 3A shows a picture of the different step wedges used. The PE, Al and Cu step wedges have nearly the same dimensions along the beam direction. Furthermore, the steel and Pb step wedges have identical dimensions along the beam direction (technical drawing provided in Supplementary Information).

Multiple radiographs were acquired with the mixed neutron and gamma-ray fission beam, each with 30 s exposure time using the three different configurations of scintillators and the Cd-B filter summarized in Table 1. The resulting images normalized by open beam after the pre-processing are shown in Fig. 3B–D. Note that a darker grey value indicates lower transmission and a grey level close to white indicates high transmission of neutrons or photons through the material.

In Fig. 3B, obtained using the Configuration #1, little difference can be observed between the transmission of the PE and Al step wedges. Comparing the Fe and Pb step wedges, they equally do not show a discernible difference in transmission. Only the Cu bloc when compared to PE and Al shows a comparatively lower transmission.

Using Configuration #2, the same step wedges show a drastically different contrast. This can be observed in Fig. 3C. Here, the transmission through PE is lower as compared to Al while Cu continues to remain opaque. A visible difference in the contrast between Pb and steel can be observed, unlike in the previous configuration. With high transmission for Al and low transmission for PE, the response of the Gadox scintillator in this configuration can be mainly attributed to that of thermal neutrons and gamma-rays.

Finally, using Configuration #3 the radiographs again show a different contrast, as shown in Fig. 3D. In this configuration, the filter efficiently shields the thermal neutrons, while allowing for gamma-rays to penetrate. As a result, the transmission of Al and PE now flips as compared to the image using the Configuration #2 (Fig. 3C). PE now shows a higher transmission compared to Al and Cu continues to remain opaque. A change in contrast is also observed between the Pb and steel sample when compared to Configuration #2, it can be observed that Pb shows a lower transmission in comparison to steel, as one would expect for gamma-rays.

From the above trends, it is evident that elemental sensitivity is enhanced if a combination of modalities are employed. This concept is further demonstrated by an example of an object constituting of different materials in the following section.

### Multimodal imaging of a high voltage cable

**Figure 4:**
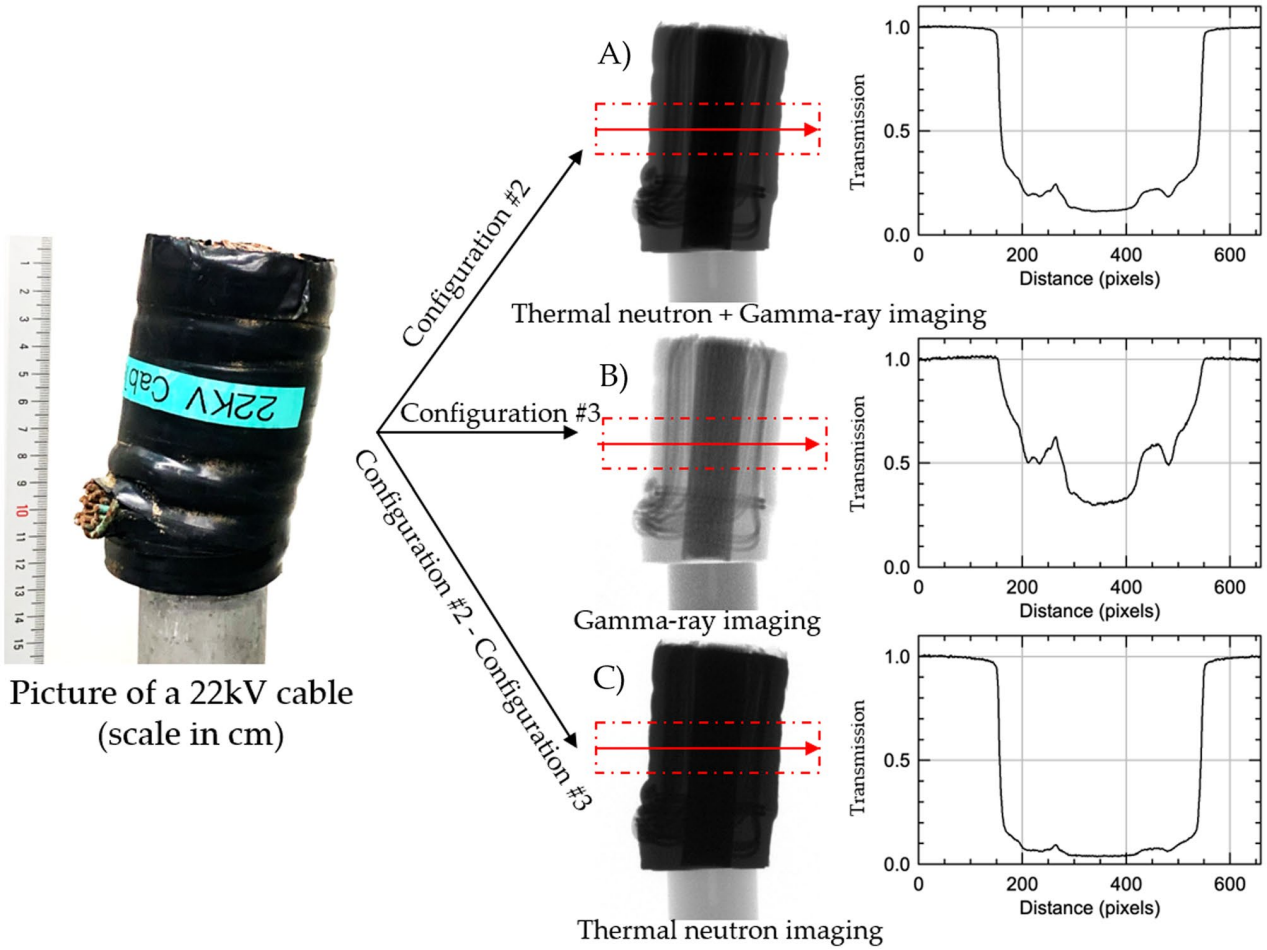
Picture of a 22 kV electric cable shown on the left. Normalized radiographs obtained using (**A**) Configuration #2, (**B**) Configuration #3 (**C**) subtraction of radiograph (**B**) from the radiograph (**A**) (Configuration #2–Configuration #3). The transmission values for the individual radiographs at positions indicated by the red arrow (averaged in height within the red dashed box) are provided on the right of each radiograph.

To demonstrate GIANT for objects constituting different materials, a 22 kV electric cable as shown on the left in Fig. 4 was imaged at NECTAR. The cable consisted of copper wires enclosed in plastic insulation and was mounted on an aluminum rod. Figure 4A, B shows the normalized radiograph of the cable with thermal neutron and gamma-ray imaging using Configuration #2 and Configuration #3. When imaged with Configuration #2, the projection consists of a mixed signals of mainly thermal neutrons and gamma-rays. The transmission values in this configuration are provided in Fig. 4A, whereby it can be observed that there is little transmission through the plastic insulation. A little over 15% transmission of signal through the cable’s insulation is observed with further reduction in transmission through the inner copper wires. The same object when imaged utilizing the Configuration #3 (Fig. 4B) provides a different contrast that can be attributed to mainly gamma-rays and in this case, the copper wires inside the plastic insulation are clearly visible, with about 50% transmission. The gamma-rays easily penetrate through the outer plastic and reveal the copper wires present inside the cable.

The radiograph in Fig. 4A is produced by a combination of thermal neutrons and gamma-rays while Fig. 4B presents mainly a gamma-ray response. Since in both radiographs, without and with the filter, the gamma contributions are nearly identical, Fig. 4C is obtained by subtracting the gamma contribution (Fig. 4B) from the neutron + gamma response (Fig. 4A), and is expected to contain mainly thermal neutron response. It should be noted that this subtraction is performed for measured and incident intensities to properly normalize the images. This resulting neutron image is shown in Fig. 4C, where less than 3% transmission through the outer plastic can be observed as indicated by the transmission profile plot on the right. Thermal neutrons cannot penetrate through the thick plastic insulation and therefore, do not provide sufficient transmission to reveal the inner copper wires of the cable. As a result, the use of the combination of two probes helps to provide a clearer understanding of an object with unknown composition.

**Figure 5:**
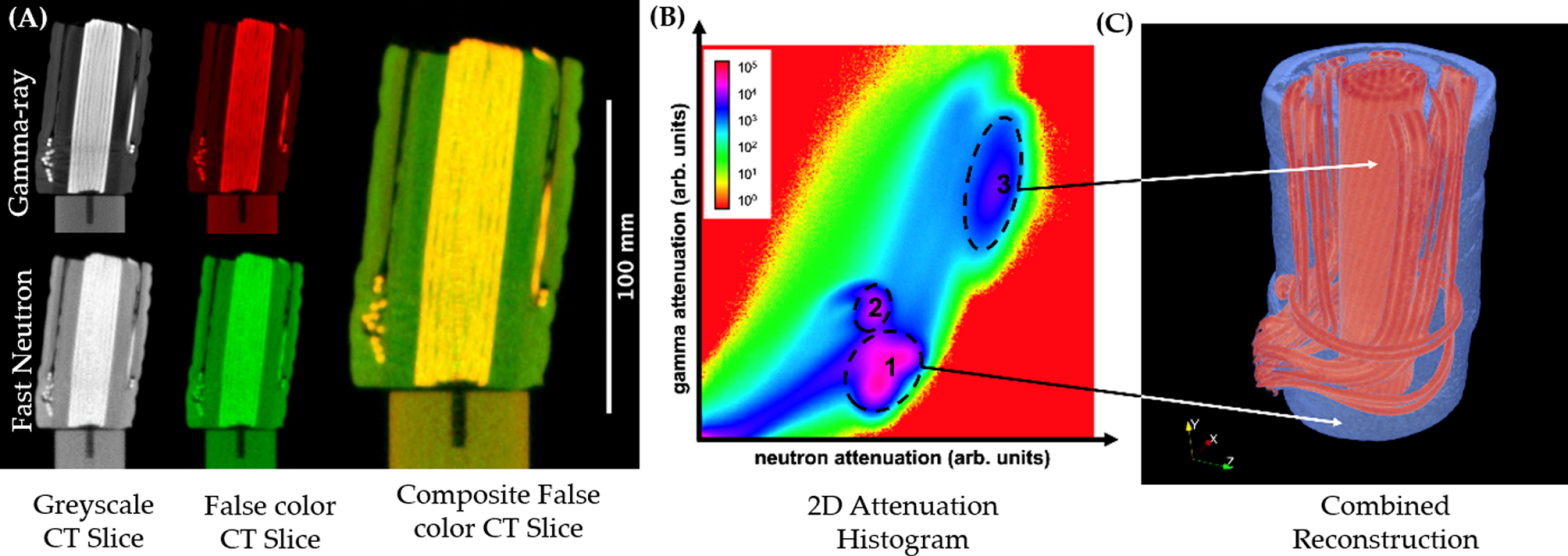
(**A**) Greyscale and false color 2D slice from reconstructed gamma-ray CT (top) and fast neutron CT (bottom) of a 22 kV cable, along with composite color CT slice of the false color slices on the right. In the images brighter areas correspond to higher attenuation and darker areas to lower attenuation, respectively. (**B**) Bivariate histogram of attenuation values for gamma-ray and fast neutron CT volumes. (**C**) 3D rendering of the regions 1 and 3 highlighted in the bivariate histogram, resulting in a combined reconstruction.

Since thermal neutrons did not provide enough penetration for the high voltage cable, fast neutron and gamma-ray CT of the same cable was performed as illustrated in Figure 5A, using Configuration #1 and Configuration #3 respectively.

A greyscale and false color 2D slice from the reconstructed tomography volume of the cable from gamma-ray and fast neutron tomography is shown in Fig. 5A, whereby the composite image of false color CT slices on the right allows easy visualization of the different imaging modalities qualitatively^24^.

Similar to other multimodal imaging applications, a bimodal histogram of the attenuation values can be used to identify the different materials or elements constituting an object^25^. Figure 5B shows a bimodal histogram of the attenuation obtained from gamma-ray and fast neutron tomography of the cable. In the histogram, three specific maxima can be observed, encircled and enumerated with 1, 2 and 3, indicating three different constituent materials present in the CT volumes. Region 1 indicates high neutron attenuation and low gamma attenuation and can be attributed to the plastic insulation. Region 2 has nearly equivalent attenuation for both, gamma and neutrons, and corresponds to the aluminum rod on which the cable was placed. Lastly, region 3 shows high attenuation for both, neutrons and gammas, and corresponds to the copper wires inside the cable. Using voxel data corresponding to region 1 and 3, volumetric rendering in blue and red color, respectively, is shown in Fig. 5C. Such analysis is helpful in differentiating the different components of an object with unknown composition, extending the capability of multimodal imaging for mm-sized objects using cold neutrons and X-rays^6,7,16,17^, to that of cm-sized objects using fast neutrons and gamma-rays.

## Discussions

To enable the identification of different materials present in a system subjected to GIANT imaging modality, the theoretical transmission values for neutrons and gammas can be calculated^26^ for different materials using the respective cross-section values from databases such as the ENDF database^27^. However, this calculation will require to take into account various factors like the exact energy spectrum of the incoming beam with or without filter, the detection efficiency of the scintillator for different energies and considerable neutron scattering corrections. While such exact values are difficult to obtain, qualitatively differentiating between the different elements present based on observed grey values is still possible as demonstrated in this work. Aside from calculating transmission values for different elements, a quantitative analysis using calibrated transmission curves for specific elements would be possible as well.

The presented GIANT approach is well suited for investigating larger samples (> 1 cm) owing to the high penetration depth of higher energy neutrons and gamma-rays, extending the capabilities for multimodal imaging of smaller samples using X-rays and cold neutrons^6,7,16,17^. Applicability of the approach presented here are for example if water intake is to be investigated in concrete blocks or rocks containing pores and cracks, whereby the multiple modalities can provide additional insight when compared to regular imaging^28^. When the cracks and pores are filled with hydrogenous substances like water, then in a neutron image these will appear as highly attenuating features and it might not be easy to categorize them as pores or cracks, while in a gamma-ray image their identification will be easier. Hence pores and cracks can be identified using gamma-rays, while for water the large neutron cross section of hydrogen provides superior contrast when compared to that of X-rays or gamma-rays. A common example in literature utilizing neutrons is studying the hydrogen uptake in scaled-up hydrogen storage systems using in situ fast neutron radiography, where the neutrons are used to study the hydrogen distribution in the storage bed upon ingress of hydrogen^14,29^. An addition of gamma imaging to such investigations can be immensely useful as this would aid in imaging of any density changes that may arise due to the presence of molten phases^29^, cracks^30^, compaction levels^31^, or design of the storage bed^32^. Similarly, the investigation of wetting of electrolyte in Li-ion batteries can also benefit from a multi-modal investigation, while the neutrons can help in revealing the distribution of electrolyte^33^, the gamma-rays can aid in revealing the inner structure and density changes in the cell. It should be noted that while GIANT is capable of imaging large systems, the resolution is inferior when compared to that of cold neutrons or X-rays. An attempt to measure the resolution was not performed in this study due to the limited beam-time available. However, features < 1 mm were identified in the radiographs (e.g. copper wires).

## Conclusions

Multimodal imaging using gamma-rays along with thermal and fast neutron imaging was performed at the NECTAR instrument of FRM II. The inherently present thermal neutrons and gamma-rays at the beamline, along with the fast neutrons were utilized for imaging by interchanging scintillators and filters. For fast neutron imaging, a PP/ZnS:Ag scintillator was used with a 2 mm Cd filter and 10 mm Borated rubber (Cd-B filter), while for gamma and thermal neutron imaging a Gadox scintillator with and without the Cd-B filter was used. Due to the same directionality of the different probes, obtained images could be directly used to compare transmitted intensities.

Using such a combination of different modalities for imaging allowed differentiation of a wide range of materials for cm-sized dense objects. This is a unique technique with many potential applications ranging from hydrogen storage materials, concrete characterization, Li-ion batteries etc., extending capabilities of cold neutrons combined with X-rays for small samples (< 1 cm) to thermal and fast neutrons in combination with gamma-rays for imaging of large objects (> 1 cm).

## Methods

### Tomography acquisition and data treatment

For gamma and neutron tomography of the electric cable, 180 projections were acquired by rotating the sample 180° in 1° steps. For each step, 4 × 30 s projections were recorded in Configuration #1 for fast neutron imaging, 1 × 30 s projections in Configuration #2 and 3 × 30 s in Configuration #3 for thermal and gamma-ray imaging, totaling in 12 h for a CT measurement of all three modalities. Additionally, 60 images each of open beam and dark field projections were also acquired with the same exposure time (30 s) for flat field normalization.

All the acquired projections (P), open beam (OB), and dark field (DF) radiographs were first pre-processed by bright spot removal and median filtering to remove gamma spots and noisy pixels. Then, the sets of open beam and dark field images were averaged and used to normalize the projections as follows:

1 $$N_{P} = \frac{P - DF}{{OB - DF}}$$

The normalized projections ($$N_{P} )$$ were then reconstructed using the SIRT reconstruction algorithm to obtain the 3D volumes. It should be noted that the axis of rotation was kept the same for all sets of data to enable a pixel-by-pixel comparison of the resulting images.

To obtain 3D volumes from thermal neutron tomography, the projections acquired in Configuration #3 were subtracted from those obtained in Configuration #2, as shown in Fig. 4. Following which, normalization and reconstruction were performed using the same procedure as described before.

### MCNP calculations

For the transport calculation, the Monte Carlo code MCNP 6.2^34^ was used. Simulations were performed with $3 \times 10^{8}$ neutrons. For all materials other than Magnesium and Calcium, cross sections from ENDF/B-VII were used. For Magnesium and Calcium, ENDF/B-VI.6 was used.

Neutron spectra shown in Fig. 1 were calculated for different combinations of input spectra and the Cd-B filter. They were recorded at the transition between beamline and MEDAPP irradiation room at a distance of 528 cm from the converter plates. A mean relative error between $11$ and $12 \%$ was achieved for all calculations. Spectra shown here should be considered as a qualitative estimate of the beam characteristics since relative errors in some energy bins were higher than recommended for MCNP results.

### Relative detection probability calculations

The energy-dependent detector efficiency $\in_{det} \left( E \right)$ and the probability $p_{ \in } \left( E \right)$ for the occurrence of the interaction used within the scintillators was calculated from the neutron fluence rates $\Phi$(E) as shown in Fig. 1 and the neutron cross sections $\sigma \left( E \right)$ for the respective interactions. For Gd and Li the spectrum with convertor and without filter (shown in red Fig. 1) was used while for hydrogen the spectrum with convertor and with filter (shown in green Fig. 1) was used.

For the calculation of a simplified energy-dependent detection probability $p_{ \in } \left( E \right)$ within the scintillators, the detector efficiency $\in_{det} \left( E \right)$ was assumed to be proportional to the product of the neutron fluence rate and mean cross section in the respective energy interval:

2 $$\in_{det} \left( {E_{mid} } \right) \propto {\Phi }\left( {E_{mid} } \right){*\,}\overline{\sigma }\left( {E_{mid} } \right)$$

Here, $E_{mid}$ indicates the mid energy of the discrete energy intervals used in the Monte Carlo simulation. For the calculation of the relative detection probability, neutron cross sections were taken from the cross section libraries BROND-2.2 for Gadolinium, CENDL-3.2 for Lithium, and ENDF/B-VIII.0 for Hydrogen. The relative detector efficiency is a measure for the number of interactions per second and could be used together with the elemental composition of the material to calculate the absolute number of interactions within the scintillators. Absolute values for the detector efficiencies are not important here since we are only interested in the interaction probability distribution. The relative interaction probability shown in Fig. 2 was calculated using the following formula:

3 $$p_{ \in } \left( {E_{mid, i} } \right) = \frac{{ \in_{det} \left( {E_{mid, i} } \right) }}{{\mathop \sum \nolimits_{i} {\Phi }\left( {E_{mid, i} } \right)* \overline{\sigma }\left( {E_{mid, i} } \right)}}$$

### Supplementary Information

Supplementary Figure S1.

## Data Availability

The raw/processed data published in this work will be provided upon reasonable request by the authors: Richi Kumar (richi.kumar@hereon.de) or Adrian S. Losko (Adrian.Losko@frm2.tum.de).
