# Supplementary material for: Gamma In Addition to Neutron Tomography (GIANT) at the NECTAR instrument
Source: Sci Rep. 2023 Nov 17;13:20120. doi: 10.1038/s41598-023-47237-y (PMC10656511; doi:10.1038/s41598-023-47237-y)
Supplement: Supplementary file 1 — Supplementary Figure S1. [file 41598_2023_47237_MOESM1_ESM.pdf]

Supplementary information

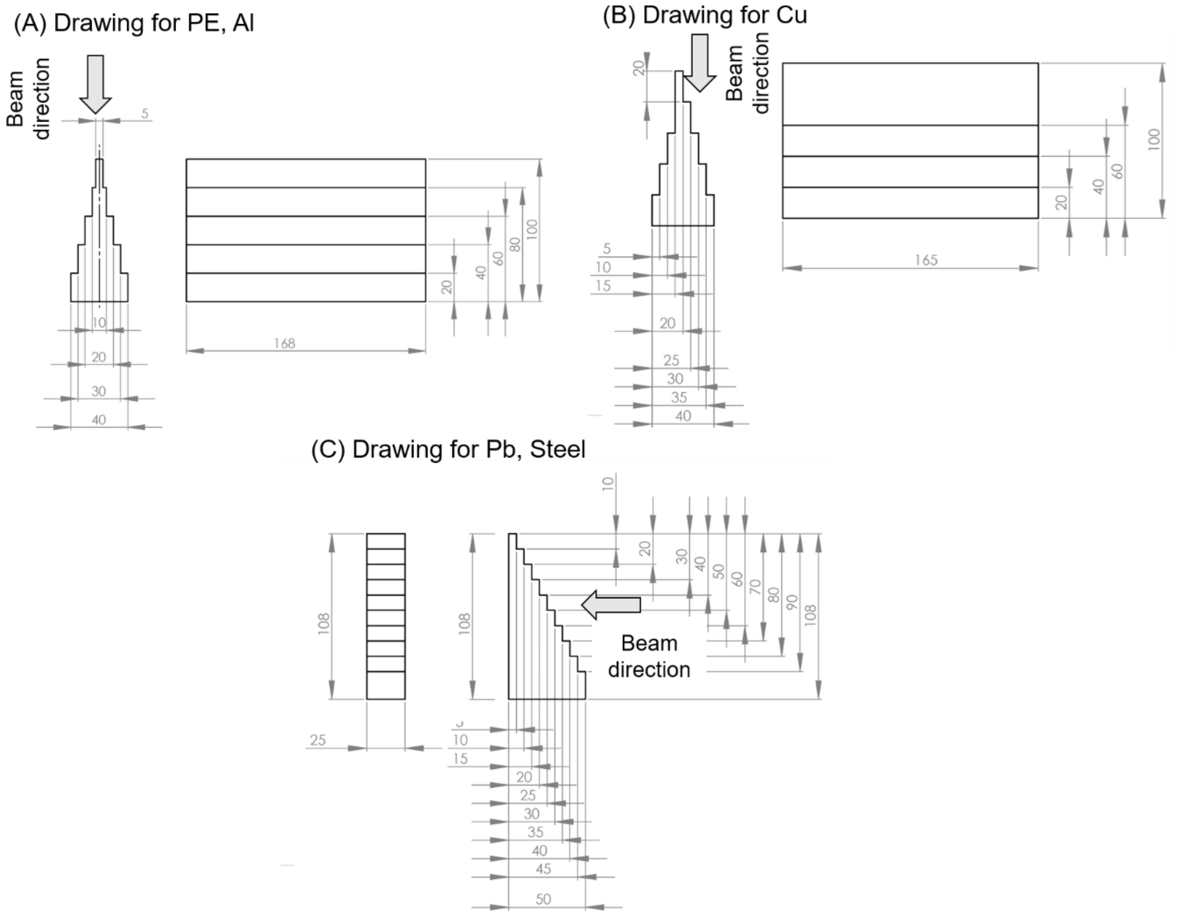

Figure S1 Drawing for the wedge of (A) Poly Ethylene (PE), Al (B) Cu and (C) Pb, Fe used for radiography in Figure 3.
